# Use of Tregs as a cell‐based therapy via CD39 for benign prostate hyperplasia with inflammation

**DOI:** 10.1111/jcmm.15137

**Published:** 2020-03-19

**Authors:** Xi Jin, Tianhai Lin, Guang Yang, Huawei Cai, Bo Tang, Xinyang Liao, Huifang Li, Xiaoting Chen, Lina Gong, Hang Xu, Yi Sun, Ping Tan, Jianqiong Yin, Hongwen Ma, Jianzhong Ai, Kunjie Wang, Qiang Wei, Lu Yang, Hong Li

**Affiliations:** ^1^ Department of Urology Institute of Urology West China Hospital Sichuan University Chengdu China; ^2^ Animal Experimental Center West China Hospital Sichuan University Chengdu China; ^3^ Laboratory of Clinical Nuclear Medicine Department of Nuclear Medicine West China Hospital Sichuan University Chengdu China; ^4^ Research Core Facility West China Hospital Sichuan University Chengdu China

**Keywords:** benign prostate hyperplasia, CD39, immunotherapy, regulatory T cells, transfusion

## Abstract

Benign prostatic hyperplasia (BPH) occurs most commonly among older men, often accompanied by chronic tissue inflammation. Although its aetiology remains unclear, autoimmune dysregulation may contribute to BPH. Regulatory T cells (Tregs) prevent autoimmune responses and maintain immune homeostasis. In this study, we aimed to investigate Tregs frequency, phenotype, and function in BPH patients and to evaluate adoptive transfer Tregs for immunotherapy in mice with BPH via CD39. Prostate specimens and peripheral blood from BPH patients were used to investigate Treg subsets, phenotype and Treg‐associated cytokine production. Sorted CD39^+/−^ Tregs from healthy mice were adoptively transferred into mice before or after testosterone propionate administration. The Tregs percentage in peripheral blood from BPH patients was attenuated, exhibiting low Foxp3 and CD39 expression with low levels of serum IL‐10, IL‐35 and TGF‐β. Immunohistochemistry revealed Foxp3+ cells were significantly diminished in BPH prostate with severe inflammatory. Although the Tregs subset was comprised of more effector/memory Tregs, CD39 was still down‐regulated on effector/memory Tregs in BPH patients. Before or after testosterone propionate administration, no alterations of BPH symptoms were observed due to CD39‐ Tregs in mice, however, CD39^+^Tregs existed more potency than Tregs to regulate prostatic hyperplasia and inhibit inflammation by decreasing IL‐1β and PSA secretion, and increasing IL‐10 and TGF‐β secretion. Furthermore, adoptive transfer with functional Tregs not only improved prostate hyperplasia but also regulated muscle cell proliferation in bladder. Adoptive transfer with Tregs may provide a novel method for the prevention and treatment of BPH clinically.

## 1 INTRODUCTION

Benign prostatic hyperplasia (BPH) occurs most commonly among older men and is often accompanied by chronic tissue inflammation.^1^ Evidence has supported the concept that age‐dependent hormonal imbalances, metabolic syndrome and chronic inflammation may be related to BPH^2^, ^3^, ^4^; however, its aetiology remains unclear. In 1987, Taguchi and Nishizuka reported that active tissue‐specific suppressor T cells participate in self‐tolerance, and their elimination induces an autoimmune response in the prostate, suggesting that autoimmune dysregulation may contribute to BPH.^5^ Regulatory T cells (Tregs) have been shown to prevent autoimmune responses and maintain immune homeostasis.^6^, ^7^ However, the role of Tregs in the pathogenesis of BPH remains the subject of intense investigation.

In several studies, Tregs have been implicated as being involved in prostate cancer and BPH.^8^, ^9^, ^10^ Ebelt et al reported that prostate cancer lesions contained numerous Tregs.^8^ In the report by Hadaschik et al, tumour‐specific effector T cells were detected in prostate cancer patients, especially in castration‐resistant prostate cancer patients; and effector T cell responses against prostate‐specific antigens increased after Treg depletion.^9^ In recent years, Davidsson et al observed a fourfold increased risk of prostate cancer in men with epithelial CD4^+^ Tregs in normal prostatic tissue, and similarly high numbers of stromal CD4^+^ Tregs were identified in post‐atrophic hyperplasia.^10^

CD39 has emerged as a marker for Tregs with potential to suppress inflammation.^11^, ^12^ Gu et al showed that human CD39^hi^ regulatory T cells manifested stronger stability and function under inflammatory conditions,^12^ and in our previous study, CD39 expression identified a subset of Tregs that displayed an effector.^13^

In this study, we aimed to investigate the Treg frequency, phenotype and function in BPH patients, and to evaluate adoptive transfer among functional Tregs for use in immunotherapy of mice with BPH.

## 2 MATERIALS AND METHODS

### 2.1 Data and sample collection

We obtained clinical data, prostatic tissues and peripheral blood samples from 38 BPH patients diagnosed with or without inflammation from West China Biobank at the West China Hospital of Sichuan University and obtained peripheral blood samples of 20 healthy male donors from KingMed Diagnostics after obtaining written informed consent. Human studies were approved by the Sichuan University Medical Ethics Committee. Specimens confirming cancer/dysplasia were excluded.

### 2.2 Flow cytometry

Isolated peripheral blood mononuclear cells (PBMCs) were used for analysis of Treg phenotypes. Cells were surface stained with fluorescently coupled antibodies specific to human antigens CD3, CD4, CD25, CD127, CD45RO, CCR7 and CD39 (purchased from BioLegend) in staining buffer followed by fixation and permeabilization (Fixation/Permeabilization Buffer, BioLegend); and intracellularly stained with fluorescently coupled anti‐Foxp3 (BioLegend) antibody.

Mouse spleen cells were isolated for CD39^+/−^ Tregs phenotypes. Cells were surface stained with fluorescently coupled antibodies specific to mouse antigens CD4, CD25, CD39, CD44, CCR7, CD62L and LAG‐3 (purchased from BioLegend) in staining buffer followed by fixation and permeabilization (Fixation/Permeabilization Buffer, BioLegend); and intracellularly stained with fluorescently coupled anti‐Foxp3 and anti‐CTLA‐4 (BioLegend) antibody.

Flow cytometric data were acquired by using a FACSAria SORP flow cytometer (BD) and analysed by using FlowJo software, version 10.5.3 (Treestar).

### 2.3 Cytokine analyses

IL‐10 (IL‐10 Human ELISA Kit, Invitrogen™), IL‐35 (Human Interleukin 35 [IL‐35] ELISA Kit, Cusabio) and TGF‐β (TGF beta‐1 Human ELISA Kit, Invitrogen™) were measured by ELISA in the serum collected from BPH patients and healthy donors, according to the manufacturer's recommendations. Mouse serum was collected to measure IL‐1β, IL‐6, TNF‐α, IL‐10, TGF‐β (purchased from Invitrogen™) and PSA (Cusabio) by ELISA according to the manufacturer's recommendations.

### 2.4 Immunohistochemistry

Human prostatic tissues were fixed in formaldehyde and embedded in paraffin, and 4‐µm paraffin sections were cut and used for immunohistochemical examination of Treg infiltration. The sections were then incubated with rabbit anti‐Foxp3 antibody (GeneTex); and after antibody binding, the sections were detected with DAB kit (Absin).

### 2.5 BPH Murine Model and anti‐CD25 or POM‐1 Administration

BABLc mice (Chengdu Dashuo experimental Animals Co., Ltd) were housed under specific pathogen‐free conditions, with temperature maintained at 20‐26ºC, and relative humidity at 40%‐70%; with free access to sterile feed and sterile water; and exposed to 12‐hour light:12‐hour dark periods in the approved Experimental Animal Center at West China Hospital of Sichuan University (Chengdu, China). Male mice, 8 to 10 weeks of age, were used for subcutaneous injection of testosterone propionate (5 mg/kg/d) for 3 consecutive weeks to establish the BPH murine model. Mice subjected to subcutaneous injection of saline were used as controls.

To deplete Tregs, mice were injected intraperitoneally with anti‐CD25 antibody (0.5 mg/d; BioXcell) on days −5, −3, and −1 before testosterone propionate administration and on days 7 and 14 after testosterone propionate administration.

To deplete CD39, CD39 activity inhibitor polyoxometalate‐1 (POM‐1; 250 μg/d; Santa Cruz Biotechnology) was intraperitoneally used on days −5, −3, and −1 before testosterone propionate administration and on days 7 and 14 after testosterone propionate administration in mice.

### 2.6 Treg isolation and cell sorting

Tregs were isolated from spleens of healthy mice using a CD4^+^CD25^+^ Regulatory T‐cell Isolation Kit (Miltenyi Biotec) according to the manufacturer's instructions. The purity of the CD4^+^CD25^+^ T cell subset was >92%. Treg cells were stained with fluorescently coupled antimouse CD39 antibody (BioLegend) and then sorted with a FACSAria SORP flow cytometer. We collected CD4^+^CD25^+^CD39^+^ and CD4^+^CD25^+^CD39^−^ cells for the assays below.

Peripheral blood mononuclear cells (PBMCs) of BPH Patients were isolated, then cells were surface stained with fluorescently coupled antibodies specific to human antigens CD3, CD4, CD25, CD127 (BioLegend), sorted by the FACSAria SORP flow cytometer. CD39^+^ Tregs or CD39^−^ Tregs were collected for the assays below.

### 2.7 In vitro suppression assays

The suppressive capacity of Treg was assessed by mixed lymphocyte reaction (MLR) according to our previous study.^14^

Carboxyfluorescein succinimidyl ester (CFSE; Invitrogen) labelled mouse peripheral blood mononuclear cells (PBMCs) as responder cells were incubated with 5 μg/mL antimouse CD3/CD28 Dynabeads (Gibco, Manufactured by Life Technologies AS) in the presence or absence of different Treg subsets in RPMI 1640 medium (Invitrogen) containing 10% foetal bovine serum for 7 days. In the coculture system, 20 μmol/L CD39 activity inhibitor polyoxometalate‐1 (POM‐1; Santa Cruz Biotechnology) was added into the MLR to evaluate the effect of CD39 on Treg‐mediated suppression. Cells were collected to analyse of CFSE dilution by flow cytometry.

Patients PBMCs as responder cells were incubated with 5 μg/mL anti‐human CD3 monoclonal antibodies (BD Pharmingen) in the presence or absence of CD39^+^ or CD39^−^ Treg subsets in RPMI 1640 medium (Invitrogen) containing 10% foetal bovine serum for 7 days. CCK8 (Dojindo) was used to detected proliferation of PBMCs according to the manufacturer's instructions.2.8 Adoptive transfer of Tregs.

Different subsets of mouse Tregs were suspended in phosphate‐buffered saline (PBS) and injected into mice intravenously (iv) at doses of 2 × 10^5^ before or after administration of testosterone propionate. Serum, prostate and bladder were collected from recipient mice at predetermined time‐points after Treg infusion to analyse cytokine secretion and lymphocytic infiltration, and for histological examination.

### 2.8 Histopathology and immunofluorescence

Fixed prostatic and bladder tissues from different groups were processed using routine paraffin techniques to prepare sections at a thickness of 4 μm. This was followed by deparaffinization, rehydration and staining with haematoxylin and eosin (H&E).

Immunofluorescent (IF) staining was used to identify the sites of CD45 and Foxp3 expression in mouse prostate tissues. Tissue sections of prostate were stained with primary antibodies CD45 (1:200, Abcam) and Foxp3 (1:200, GeneTex). Then, sections were stained with the fluorescent secondary antibodies Alexa Fluor^®^ 488 donkey anti‐rabbit IgG antibody (Abcam) and Alexa Fluor^®^ 594 donkey antimouse IgG antibody (Abcam); and with 4′6‐diamidino‐2‐phenylindole (DAPI, Sigma‐Aldrich Co., Ltd). Sections were then mounted with antifade mounting medium (Abcam).

### 2.9 Statistical analysis

The distributions of Treg‐associated markers of values for BPH and healthy control populations were tested using the Kolmogorov‐Smirnov test. We used two‐tailed Student *t* test to compare BPH patients and healthy controls. The unpaired *t* test with Welch's correction was used when the KS test was not statistically significant, while the Mann‐Whitney U test was used when the KS test was found to be statistically significant. One‐way ANOVAs followed by a multiple‐comparison test such as Tukey's test was used to compare among different mice groups. Results were presented as means ± SD *P < *.05 was considered to be significant.

## 3 RESULTS

### 3.1 Characteristics of the study population

We included a total of 38 patients with BPH and 20 healthy controls in this study. Clinical characteristics of BPH patients are shown in Table S1. The mean age of patients with BPH was 67.05 ± 8.04 years (mean ± SD), and the range of age in healthy controls was from 30 to 50 years. The BPH patients were divided by inflammation into two groups according to pathologic diagnosis. The mean PSA values for patients with BPH and inflammation vs patients with BPH alone were 26.43 ± 27.79 ng/mL and 10.96 ± 5.76 ng/mL, respectively (*P < *.05). The mean fPSA values for patients with BPH and inflammation and patients with BPH alone were 4.09 ± 6.50 ng/mL and 2.28 ± 2.58 ng/mL, respectively.

### 3.2 Treg subsets in BPH patients

**Figure 1:**
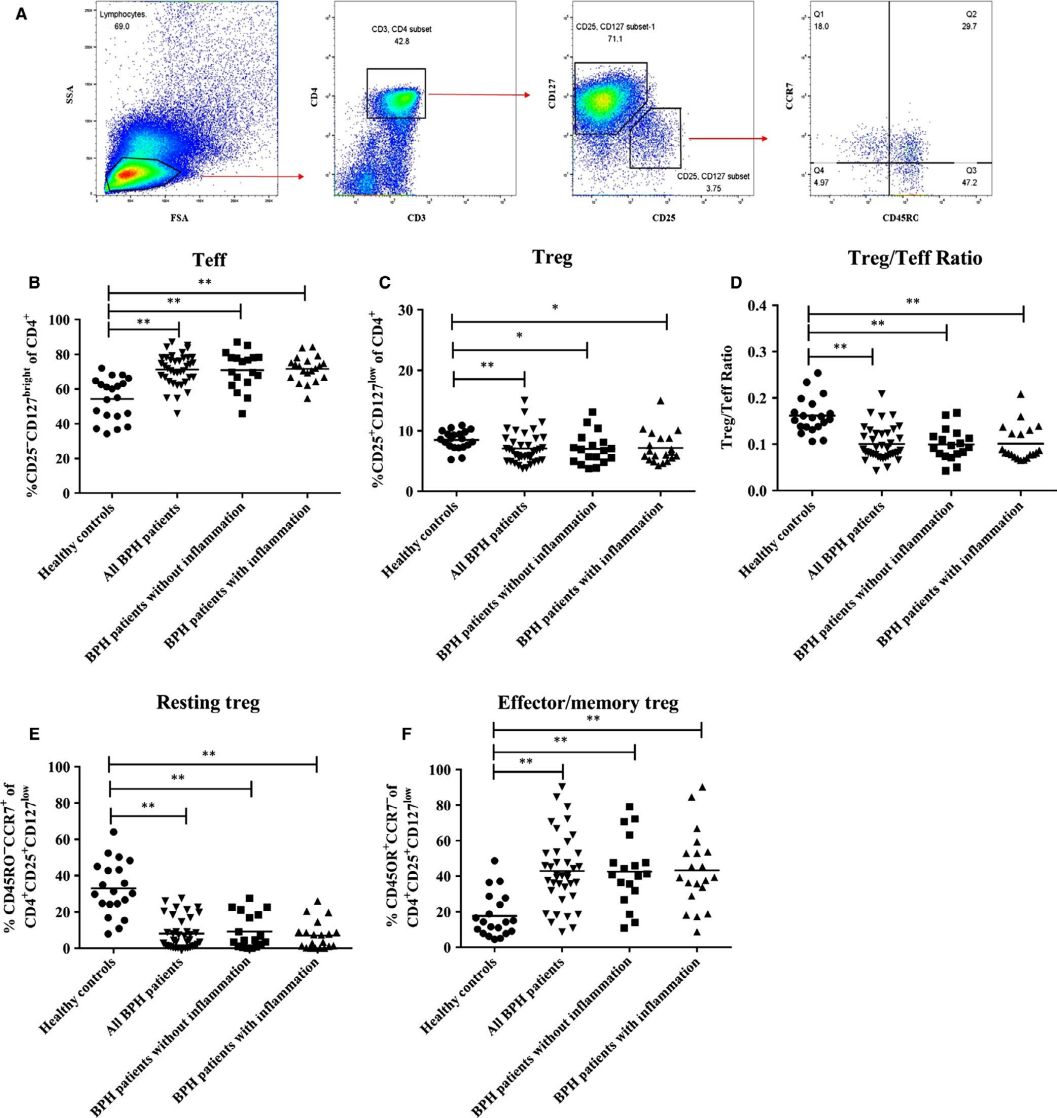
Frequency of Treg subsets in BPH patients and healthy controls. A, Representative FACS analysis shows the gating strategy to identify CD3^+^CD4^+^CD25^+^CD127^−^CD45RO^+/−^CCR7^+/−^ in peripheral blood of BPH patients. Lymphocytes were first gated on CD3‐ and CD4‐positive cells, and then gated on CD25‐positive, CD127‐negative cells. Finally, cells were used with CD45RO and CCR7 to differentiate between resting and effector/memory Treg subsets. B, Analysis of isolated PBMCs from healthy controls and BPH patients for the frequency of Teffs (CD3^+^CD4^+^CD25^−^CD127^high^). C, Analysis of isolated PBMCs from healthy controls and BPH patients for the frequency of Tregs (CD3^+^CD4^+^CD25^+^CD127^l^°^w^). D The ratio of Tregs to Teff percentages in healthy controls and BPH patients. E, Analysis of isolated PBMCs from healthy controls and BPH patients for the frequency of resting Tregs (CD3^+^CD4^+^CD25^+^CD127^l^°^w^CD45RO^−^CCR7^+^). F, Analysis of isolated PBMCs from healthy controls and BPH patients for the frequency of effector/memory Tregs (CD3^+^CD4^+^CD25^+^CD127^l^°^w^CD45RO^+^CCR7^−^). These are *P* values. *P < 0.05 and **P < 0.01

To analyse subsets of Tregs, CD3^+^CD4^+^T cells were gated from the lymphocyte gate. Then Tregs were gated on CD25^+^CD127^low^ cells, and effector T cells (Teffs) were gated on CD25^−^CD127^birhgt^. Furthermore, in CD4^+^CD25^+^CD127^l^°^w^ cell subsets (Tregs), resting Tregs were gated on CD45RO^−^CCR7^+^ and effector/memory Tregs were gated on CD45RO^+^CCR7^−^ (Figure 1A).

In BPH patients with or without inflammation, Teffs were significantly higher in peripheral blood than in healthy controls (*P < *.01, Figure 1B), while Tregs were significantly lower than in healthy controls (*P < *.05*,* Figure 1C); and we calculated a significant reduction in the Treg/Teff ratio compared to healthy controls (*P < *.01, Figure 1D). To analyse Tregs subsets, CD45RO and CCR7 were used to differentiate resting Tregs from effector/memory Tregs. Tregs contained a smaller percentage of resting Tregs in BPH patients when compared to healthy controls (*P < *.01, Figure 1E), while BPH patients showed a higher percentage of effector/memory Tregs than did healthy controls (*P < *.01, Figure 1F).

### 3.3 Treg function was impaired in the BPH patients

**Figure 2:**
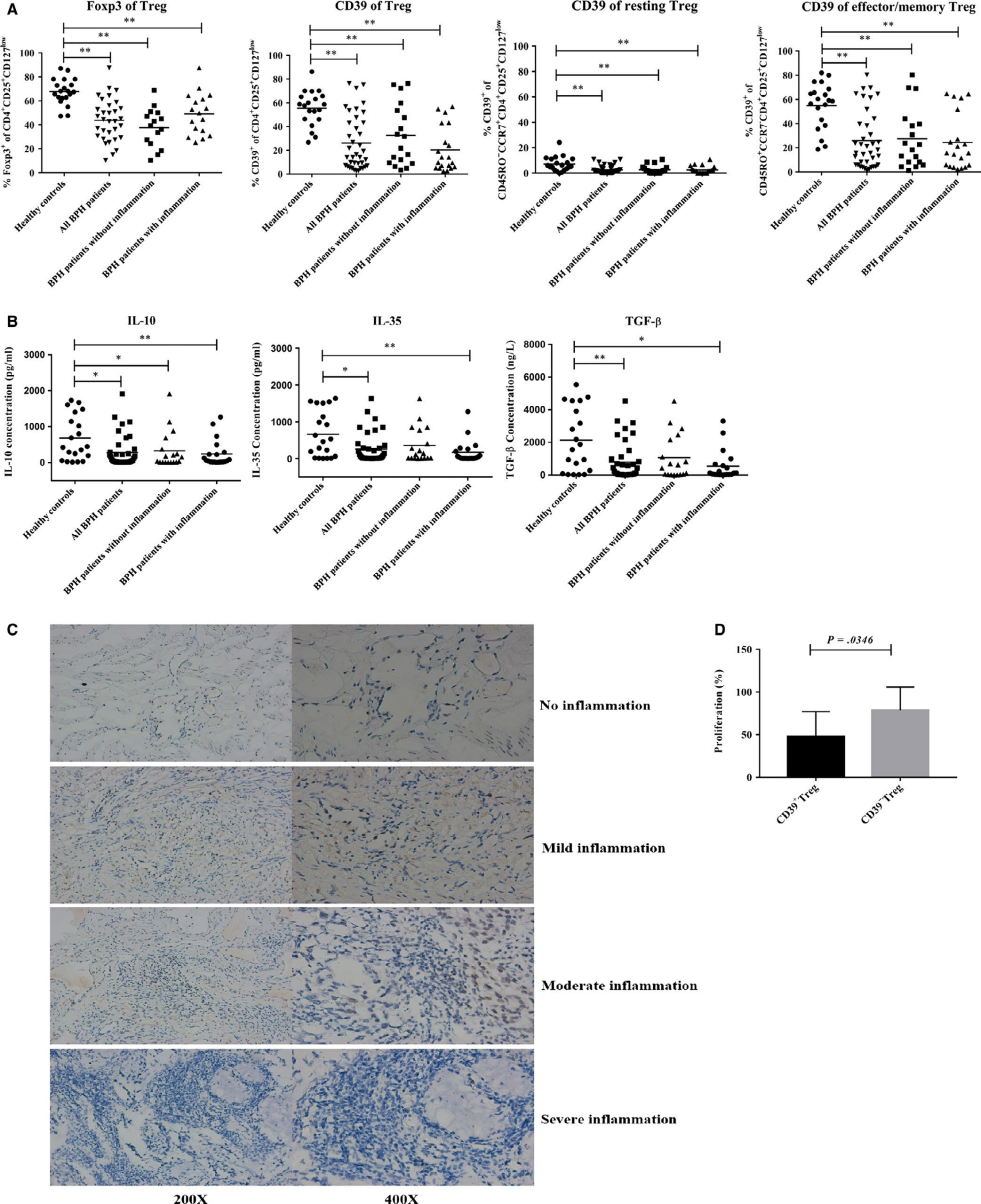
Characteristics of functional markers on Treg subsets in BPH patients. A, PBMCs from healthy controls and BPH patients were surface‐ or intracellularly stained with anti‐human CD39 or Foxp3 antibody, and flow cytometric data were analysed for the frequency of Foxp3^+^ and CD39^+^ on total Tregs, resting Tregs and effector/memory Tregs. All cells were gated on CD3^+^T cells. B, We used ELISAs to determine cytokine concentrations of IL‐10, IL‐35 and TGF‐β in serum collected from healthy controls and BPH patients. C, Representative immunohistochemical images of Foxp3 staining in prostate. BPH patients were divided into groups with or without inflammation. BPH patients with inflammation were divided into additional groups according to the grade of inflammation. Brown dots represent Foxp3^+^ cells in BPH patients with no inflammation (200×; 400×), BPH patients with mild inflammation (200×; 400×), BPH patients with moderate inflammation (200×; 400×) and BPH patients with severe inflammation (200×; 400×). D, In vitro suppression assay of sorted CD39^+/−^ Tregs subsets from BPH patients by mixed lymphocyte reaction. Patients peripheral blood mononuclear cells (PBMCs) as responder cells were incubated with 5 μg/mL anti‐human CD3 monoclonal antibodies in the presence or absence of CD39^+^ or CD39^−^ Treg subsets for 7 days. CCK8 was used to detected proliferation of PBMCs. Data are mean ± SD, *P < .05 and **P < .01

To test the function of Tregs among different groups, we evaluated Foxp3 and CD39 expression and serum cytokines, and observed that BPH patients with or without inflammation showed lower levels of Foxp3 expression compared to healthy controls (*P < *.01, Figure 2A); and the percentage of CD39^+^ Tregs was also lower in BPH patients (*P < *.01, Figure 2A). Although Tregs contained a higher percentage of effector/memory Tregs in BPH patients than in healthy controls (Figure 2A), CD39 expression on effector/memory Tregs was still lower in BPH patients (Figure 2A). Interestingly, CD39 was rarely expressed on resting Tregs, either in healthy controls or BPH patients (Figure 2A). This suggested that CD39 was important in the functioning of the effector/memory Treg subset.

We assessed immunosuppressive cytokines in serum (representative of Treg suppression, such as IL‐10, IL‐35 and TGF‐β) by ELISA and demonstrated that IL‐10 levels in healthy controls were higher than in BPH patients with or without inflammation (*P < *.05, Figure 2B). IL‐35 and TGF‐β levels were also significantly lower in BPH patients with inflammation relative to healthy controls (*P < *.05, Figure 2B). These results suggested that Tregs secreted fewer suppressive cytokines in patients with BPH.

When we observed Treg infiltration into prostatic tissue and detected Foxp3‐positive cells by IHC, we noted a small number of Foxp3^+^ cells in the non‐inflammatory prostate tissue (Figure 2C); but Foxp3^+^ cells increased in the tissue of BPH patients with mild or moderate inflammation (Figure 2C). In patients with severe inflammatory BPH, a large number of inflammatory cells infiltrated into the prostatic tissue, but the number of Foxp3^+^ cells decreased significantly (Figure 2C). These results suggested that an altered frequency and impaired function of Tregs occurred in BPH patients.

To demonstrate the significance of CD39 on Tregs, CD39^+^ or CD39^−^ Tregs were sorted to assess in vitro suppressive capacity using the MLR assay. The CD39^+^ Tregs sorted from BPH patients showed more potent of suppressive capacity than CD39^−^ Tregs, as demonstrated by inhibition of responder cell proliferation at a ratio of responder cells: Tregs of 10:1 (proliferation of CD39^+^Tregs 47.96 ± 27.51% vs proliferation of CD39^−^ Tregs 78.66 ± 25.61%, Figure 2D). These results suggest that CD39 is involved in the suppression of Tregs.

### 3.4 Anti‐CD25 contribute to the progression of BPH

**Figure 3:**
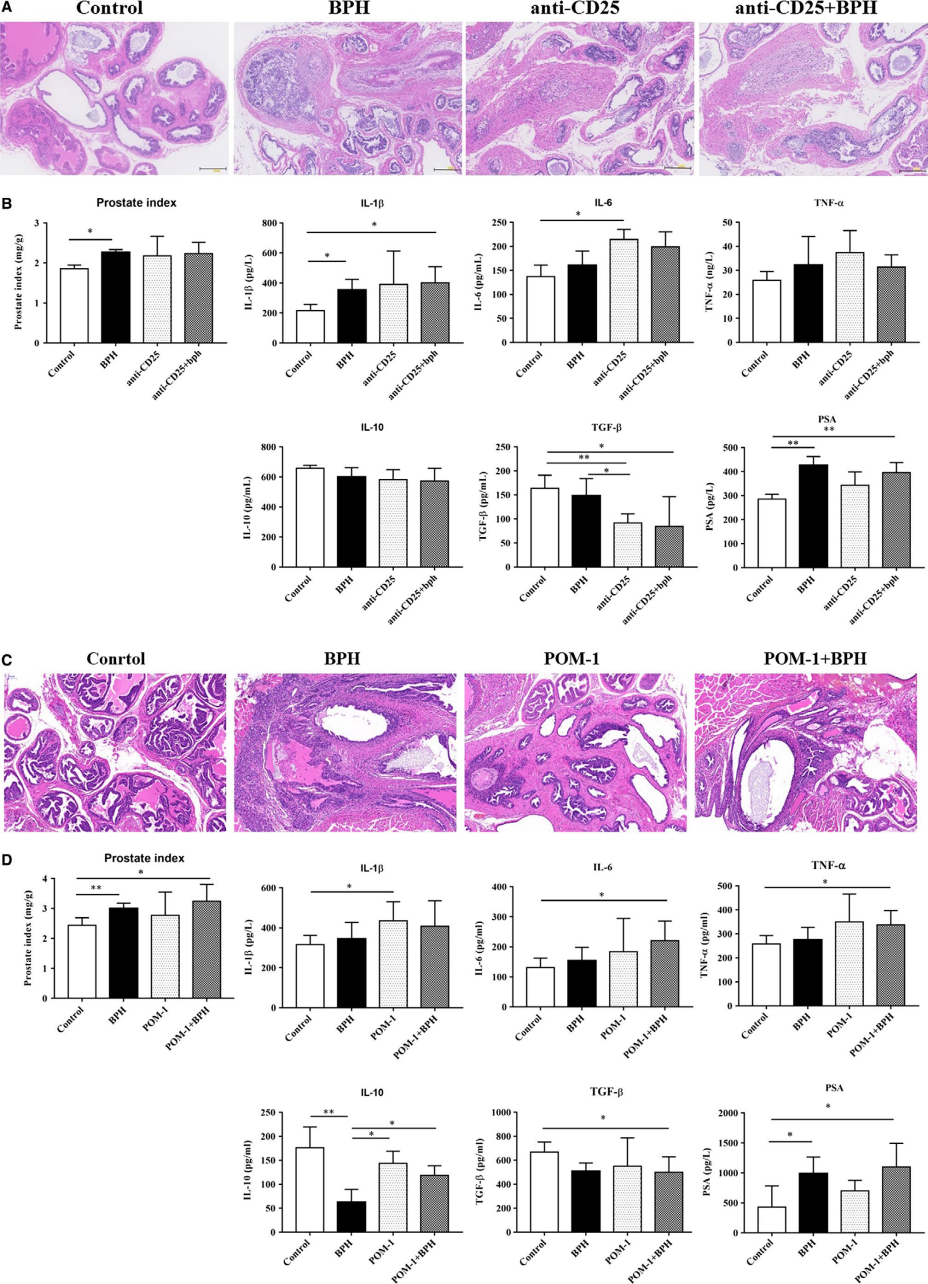
Mouse prostate and serum cytokines collected from mice administered anti‐CD25 antibody or POM‐1 with the administration of testosterone propionate. A, representative haematoxylin‐eosin staining of mouse prostate. B, mouse prostate indices in different groups were calculated as study end‐points. Prostate index (mg/g) = prostate weight/body weight. Mouse serum was collected at the study end‐points, and cytokine concentrations of IL‐1β, IL‐6, TNF‐α, IL‐10, TGF‐β and PAS in serum were evaluated by ELISA. C, representative haematoxylin‐eosin staining of mouse prostate. D, mouse prostate indices in different groups were calculated as study end‐points. Mouse serum was collected at the study end‐points, and cytokine concentrations of IL‐1β, IL‐6, TNF‐α, IL‐10, TGF‐β and PAS in serum were evaluated by ELISA. Data are mean ± SD of three independent experiments. *P < .05 and **P < .01

To validate the role of Tregs in BPH, mice were injected intraperitoneally with anti‐CD25 antibody before and after testosterone propionate administration to deplete Tregs. After anti‐CD25 antibody administration, we found stromal cell hyperplasia and epithelial cell hyperplasia in the prostate with or without testosterone administration (Figure 3A). The prostatic index of mice was increased after anti‐CD25 antibody administration with significantly increased IL‐1β, IL‐6 and PSA secretion and decreased TGF‐β secretion (Figure 3B). These results suggested that depletion of Tregs may lead to prostate hyperplasia with inflammation.

### 3.5 Anti‐CD39 facilitate to the progression of BPH

To confirm the significance of CD39^+^ Treg cells in progression of BPH, mice were injected intraperitoneally with CD39 activity inhibitor polyoxometalate‐1 (POM‐1) before and after testosterone propionate administration. After POM‐1 administration, we found stromal cell hyperplasia and epithelial cell hyperplasia in the prostate with or without testosterone administration (Figure 3C). The prostatic index of mice was increased after POM‐1 administration with significantly increased IL‐1β, IL‐6, TNF‐α and PSA secretion and decreased IL‐10 and TGF‐β secretion (Figure 3D). These results indicated that depletion of CD39 may facilitate to the progression of BPH.

### 3.6 Transfer of Tregs to mice controls inflammation and the development and progression of BPH via CD39

**Figure 4:**
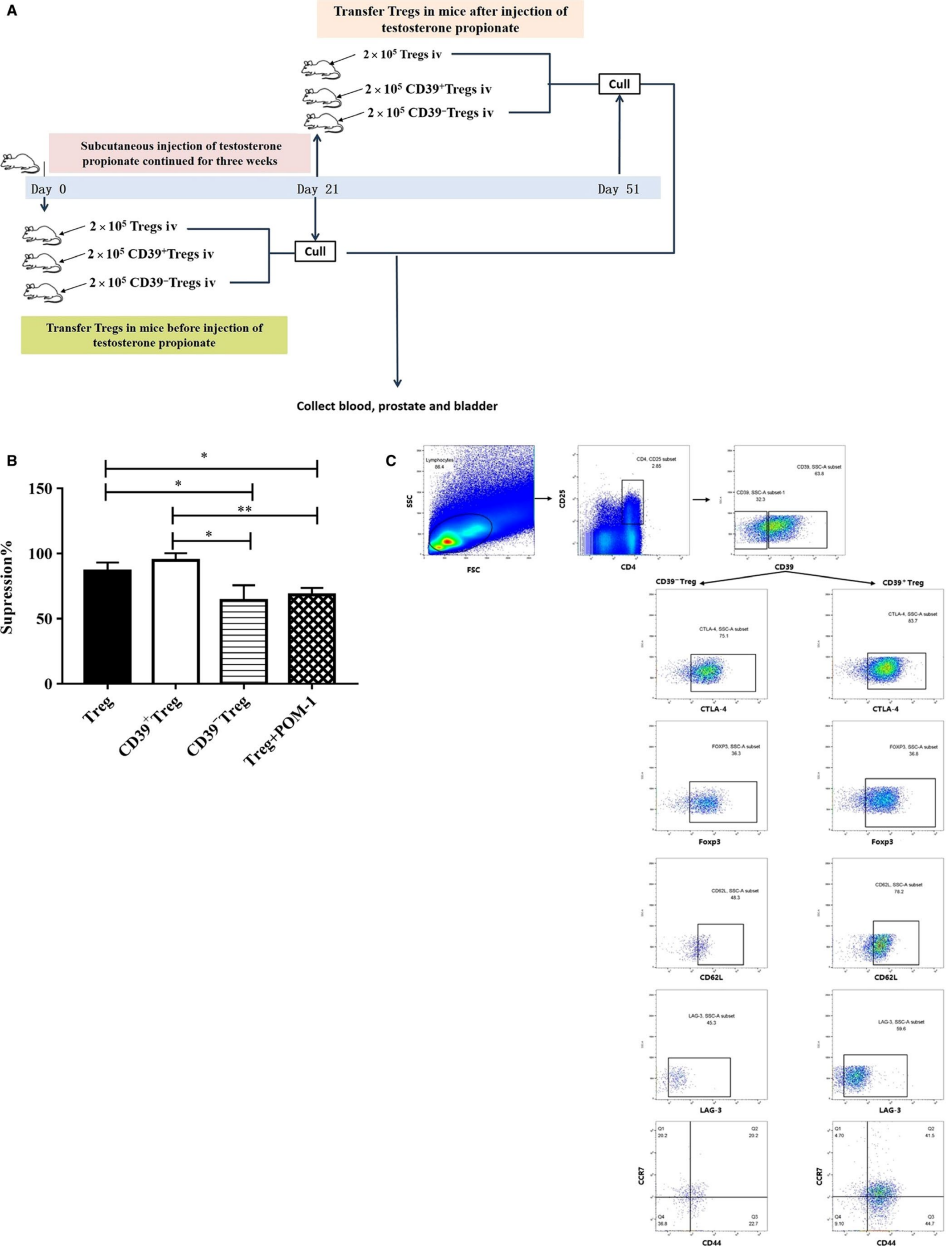
The characterization of CD39^+^ and CD39^−^ Treg cells in vitro. A, schematic representation of the in vivo transferred Tregs in the mouse model. B, In vitro suppression assay of sorted murine Tregs subsets by mixed lymphocyte reaction. Carboxyfluorescein diacetate succinimidyl ester (CFSE)‐labelled mouse PBMCs were stimulated with antimouse CD3 mAb as responder cells in the presence or absence of Treg subsets with or without CD39 activity inhibitor POM‐1 for 7 d prior to measurement of PBMCs proliferation by CFSE dilution. C, representative flow cytometric analysis of phenotype of CD39^+^ and CD39^−^ Tregs. Gates were set on CD4^+^CD25^+^ cells, CD39^−^ or CD39^+^ cells were sorted to detect Treg phenotype, such as Foxp3, CTLA‐4, CD62L and LAG‐3. Resting Tregs were gated as CD44^−^CCR7^+^, effector Tregs were gated as CD44^+^CCR7^−^, and memory Tregs were gated as CD44^+^CCR7^+^. These are *P* values. *P < 0.05 and **P < 0.01

To identify the role of Tregs in development and progression of BPH, Tregs and CD39^+/−^ Treg subsets sorted by flow cytometry were transferred into mice before or after the daily injection of testosterone propionate for 3 weeks to establish the BPH murine model (Figure 4A). Firstly, suppressive capacity of sorted Tregs was detected in vitro MLR assays and adding the CD39 activity inhibitor (POM‐1) into the MLR was to validate the important role of CD39 in Tregs suppression. Sorted Tregs and CD39^+^Treg maintained their potency to suppress the proliferation of responder cells (suppression at 86.63 ± 6.62%, 94.73 ± 5.67%, respectively, Figure 4B). However, suppressive potency of CD39^−^ Treg and Treg with POM‐1 treatment was reduced in MLR (suppression at 63.95 ± 11.69%, 68.26 ± 5.45%, respectively, Figure 4B). The results suggested that CD39 was involved in the Tregs suppressive capacity.

Secondly, the phenotype of CD39^+/−^ Treg subsets from spleen was analysed by flow cytometry. CD39^+^ Tregs expressed high levels of CTLA‐4 (76.6 ± 7.1% of CD39^+^Tregs vs 69.2 ± 5.9% of CD39^−^ Tregs, Figure 4C), CD62L (80.8 ± 2.6% of CD39^+^Tregs vs 44.9 ± 3.4% of CD39^−^ Tregs, *P < *.05*,* Figure 4C) and LAG‐3 (60.9 ± 1.3% of CD39^+^Tregs vs 43.2 ± 2.4% of CD39^−^ Tregs, *P < *.05*,* Figure 4C). Interestingly, CD39^+^ Tregs showed more effector Treg phenotype (CD44^+^CCR7^−^, 49.9 ± 5.2% of CD39^+^Tregs vs 17.8 ± 4.9% of CD39^−^ Tregs, *P < *.05*,* Figure 4C) than CD39^−^ Tregs, but less resting Treg (CD44^−^CCR7^+^, 5.6 ± 0.9% of CD39^+^Tregs vs 19.9 ± 0.4% of CD39^−^ Tregs, *P < *.05*,* Figure 4C) phenotype than CD39^−^ Tregs.

**Figure 5:**
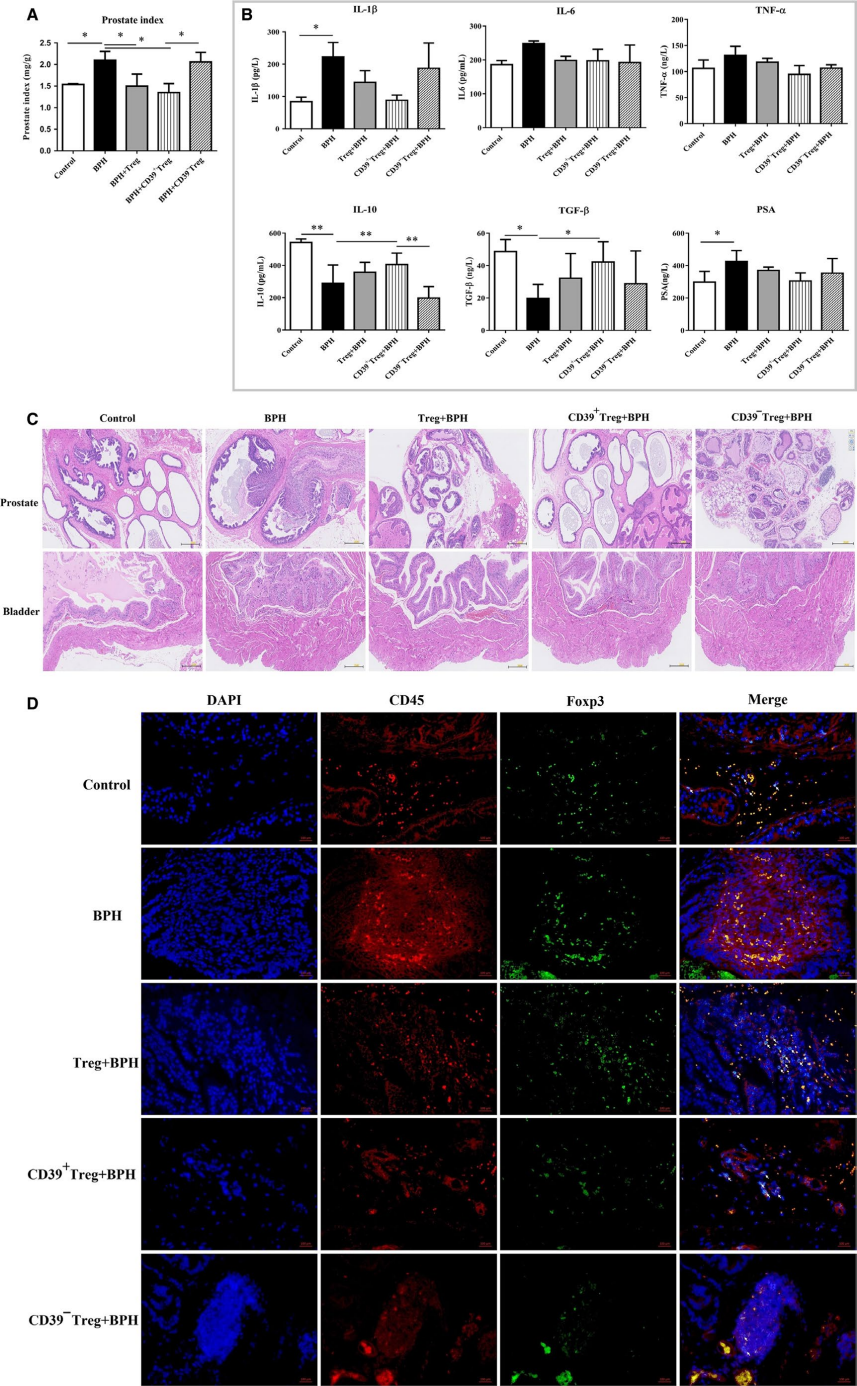
Mouse prostate index, serum cytokines, prostate and bladder collected from mice administered Treg subsets before testosterone propionate administration, A, Mouse prostate indices in different groups were calculated at study end‐points. Prostate index (mg/g) = prostate weight/body weight. B, Mouse serum was collected at study end‐points, and cytokine concentrations of IL‐1β, IL‐6, TNF‐α, IL‐10, TGF‐β and PAS in serum were detected by ELISA. Data are mean ± SD of three independent experiments. *P < .05 and **P < .01*.* C, Prostate and bladder of control mice, BPH mice, Treg‐infused mice, CD39^+^ Treg‐infused mice and CD39‐Treg‐infused mice were collected for histological examination using haematoxylin‐eosin staining. D, Representative images of immunofluorescence staining of immune cells. Native PE (CD45, red) and FITC (Foxp3, green) fluorescence images and merged images with DAPI staining (blue) of the same sections are also shown. White arrows indicate representative functional Tregs

After testosterone administration, the prostatic index of mice was increased, and we found stromal cell hyperplasia and epithelial cell hyperplasia with inflammatory cell infiltration in the prostate (Figure 5A,C).

Prior to the injection of testosterone propionate, transfer of CD39^+^Tregs has more potent to control the prostate index than CD39^−^ Tregs (*P < *.05*,* Figure 5A). Transfer of Tregs and CD39^+^Tregs decreased IL‐1β and PSA secretion and increased IL‐10 and TGF‐β secretion (Figure 5B). Treg transfusion alleviated prostate hyperplasia and inflammation, but prostate cells showing deformation and necrosis were still found in the prostate (Figure 5C), and Foxp3^+^cells were found around the inflammatory cells (Figure 5D). CD39^−^ Treg transfer did not change symptoms of BPH in mice (Figure 5C), and we rarely observed Foxp3^+^ cell infiltration into the prostate around inflammatory cells (Figure 5D). However, transferred CD39^+^Treg controlled prostate hyperplasia (Figure 5B) and inhibited inflammation by increasing Foxp3^+^ cell infiltration (Figure 5D).

**Figure 6:**
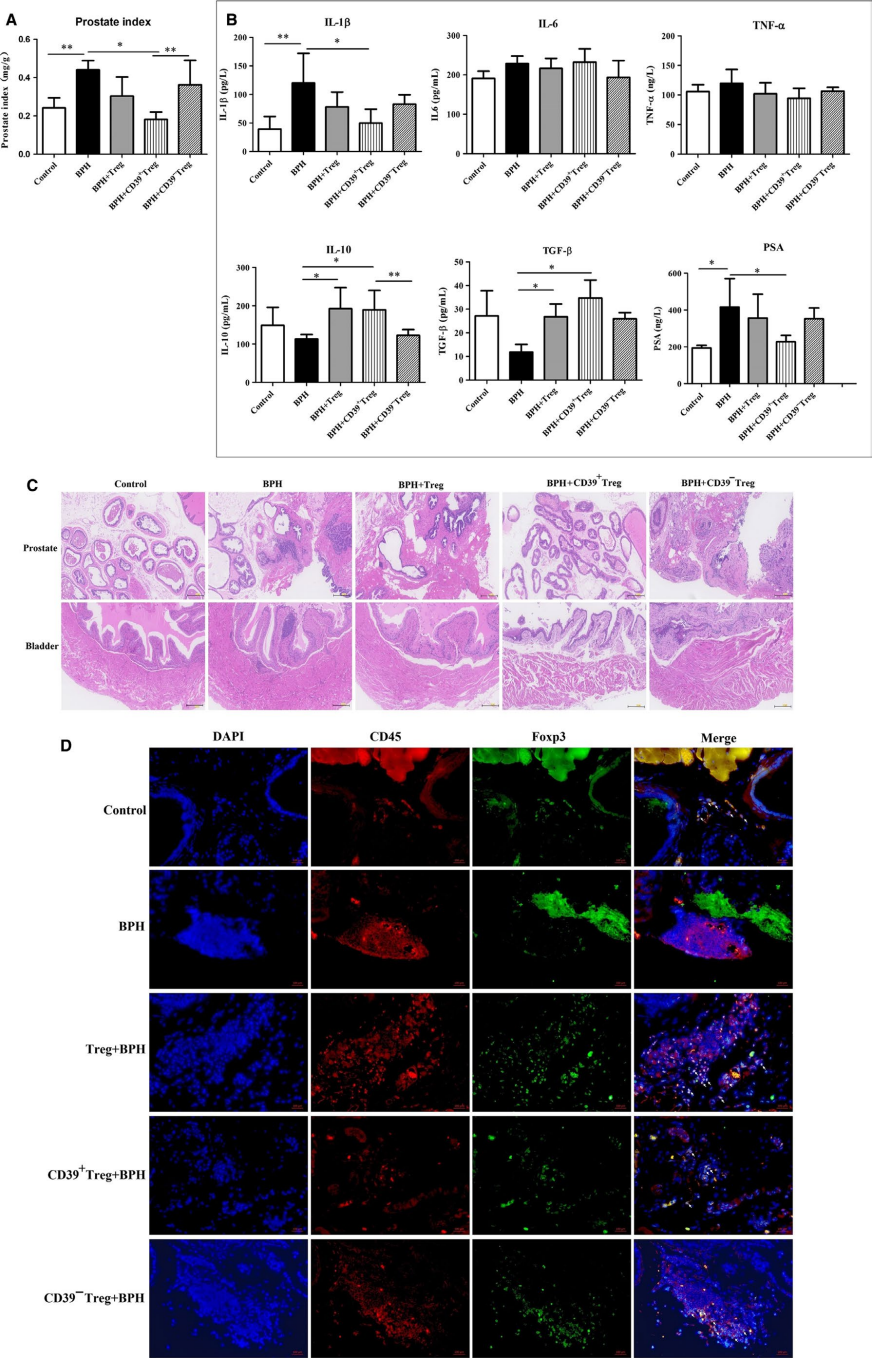
Mouse prostate index, serum cytokines, prostate and bladder collected from mice administered Treg subsets after testosterone propionate administration. A, Mouse prostate indices in different groups were calculated at study end‐points. Prostate index (mg/g) = prostate weight/body weight. B, Mouse serum was collected at study end‐points, and cytokine concentrations of IL‐1β, IL‐6, TNF‐α, IL‐10, TGF‐β and PAS in serum were detected by ELISA. Data are mean ± SD of three independent experiments. *P < .05 and **P < .01*.* C, Representative haematoxylin‐eosin staining of mouse prostate and bladder images from Treg subsets administered to mice after testosterone propionate administration. D, Representative images of immunofluorescence staining of immune cells. Native PE (CD45, red) and FITC (Foxp3, green) fluorescence images, and merged images with DAPI staining (blue) for the same sections are also shown. White arrows indicate representative functional Tregs

We then transferred different Treg subsets into mice after injection of the testosterone propionate. CD39^+^Treg infusion significantly decreased the prostate index in mice more than CD39^−^ Treg (*P < *.01*,* Figure 6A), reduced IL‐1β and PSA secretion (*P < *.05*,* Figure 6B), and increased IL‐10 and TGF‐β secretion (Figure 6B). After Treg transfusion, stromal cell hyperplasia and epithelial cells deformation and necrosis were still found in mouse prostates (Figure 6C), and Foxp3^+^cells were found around inflammatory cells (Figure 6D). Consistent with Treg transfusion before testosterone propionate injection, transfer of CD39^−^ Tregs did not improve BPH (Figure 6C), and Foxp3^+^ cells were rarely observed around inflammatory cells in the prostate (Figure 6D). Nevertheless, transferred CD39^+^Tregs alleviated prostate hyperplasia (Figure 6C) and inhibited inflammation by increasing Foxp3^+^ cell infiltration (Figure 6D).Interestingly, adoptive transfer with functional Tregs not only improved hyperplasia and inflammation in the prostate, but also controlled muscular proliferation in the bladder (Figures 5A and 6C).

**Figure 7:**
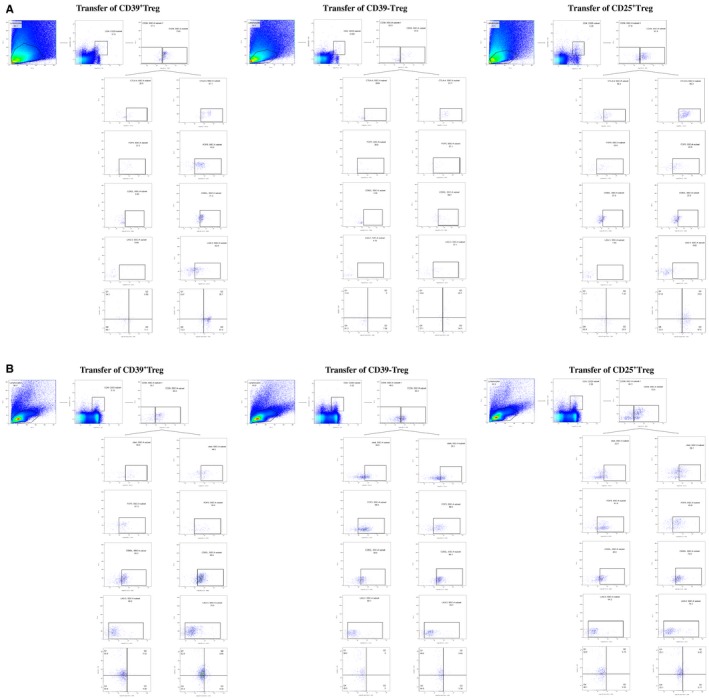
Phenotype characteristics of CD39^+/−^ Tregs in prostate (A) and peripheral blood (B) after Tregs infusion. Tregs were gated on CD4^+^CD25^+^ cells, and then phenotype characteristics were gated on CD39^+/−^ Tregs. In the CD39^+/−^ Treg cell subsets, resting Tregs were gated as CD44^−^CCR7^+^, effector Tregs were gated as CD44^+^CCR7^−^, and memory Tregs were gated as CD44^+^CCR7^+^ cell

In addition, the phenotype of CD39^+/−^ Treg subsets from prostate tissue and peripheral blood was detected by flow cytometry after mouse infusion of different subsets of Tregs. After CD39^+^Tregs transferred, more CD39 positive Tregs, which expressed high level of CTLA‐4, Foxp3 and CD62L, were found in prostate than CD39^−^ Tregs and CD25^+^Tregs transferred mouse (Figure 7A, Table S2). In prostate, CD39^+^Tregs exhibited phenotype of effector or memory Tregs, in other hand, CD39^−^ Tregs almost did not show effector or memory Tregs phenotypes (Figure 7A, Table S2). However, in peripheral blood, CD39^+^Tregs infusion did not elevate the expression of CTLA‐4, Foxp3 and CD62L in CD39 positive Tregs when compared to CD39^−^ Tregs or CD25^+^Tregs infusion (Figure 7B, Table S2). Further, CD39^+^ or CD39^−^ Tregs showed more resting Tregs phenotype in peripheral blood than in prostate (Figure 7B, Table S2). These results suggested that transferred CD39^+^Tregs could alter Treg phenotypes into functional and effector/memory Treg phenotypes to acquire more potent to suppress in pathological tissue.

## 4 DISCUSSION

Our data show impaired function of Tregs in BPH patients due to attenuated expression of CD39. Transferred Tregs in mice before or after injection of testosterone propionate not only inhibited prostate inflammation and ameliorated prostate hyperplasia but also controlled thickening of the bladder muscular layer via CD39.

CD39 is an ectoenzyme that hydrolyses adenosine triphosphate and adenosine diphosphate to adenosine monophosphate (AMP) and exhibits immunosuppressive effects.^15^, ^16^ Growing evidence implicates an important role for CD39 in Treg‐suppressive function.^17^, ^18^, ^19^ In our previous study of diabetes, the defective suppressive function of Tregs in type 1 diabetic patients is due to lowered expression of CD39 on Tregs.^13^ In this study, we also observed CD39 expression on Tregs to be lower in BPH patients. Intriguingly, we found that CD39 is rarely expressed on the resting subset of Tregs, but expression on the effector/memory Treg subset indicated that CD39 may play a role in the function of effector/memory Tregs. Then, CD39 activity inhibitor polyoxometalate‐1 (POM‐1) was used in mice before and after testosterone propionate administration to confirm the significance of CD39 in BPH. We found that depletion of CD39 may facilitate to the progression of BPH. Furthermore, CD39^+^Tregs sorted from healthy mice were more potent than Tregs in protecting prostate and bladder, suggesting that the CD39^+^Treg subset plays an important role in BPH.

Tregs are essential for the maintenance of peripheral self‐tolerance, and this has stimulated strong interest in their potential therapeutic application in ameliorating autoimmune diseases.^20^ The interleukin‐2 receptor α chain (IL‐2Rα, CD25) plays a major role in shaping the dynamics of T cell populations following immune activation. Numerous previously published studies have used anti‐CD25 antibody to deplete Treg cells. The results showed that anti‐CD25 antibody induces deletion of Tregs markers.^21^, ^22^, ^23^ Anti‐CD25 antibody can reduce almost all CD4^+^CD25^+^cells and has less effect on CD4^+^CD25^−^cells both in vitro and in vivo studies.^24^ However, there was a study to report that graft biopsies showed that basiliximab therapy which directly target CD25 leads to high expression of Foxp3 locally in the graft after kidney transplantation.^25^ Cai et al reported that short‐term treatment with anti‐CD25 antibody might not influence Tregs.^26^ These may indicate that Tregs could be induced by CD4^+^CD25^−^ T cells. Induced CD4^+^Foxp3^+^ Tregs could be generated from CD4^+^CD25^−^ T cells which represent another subset of CD4^+^ Tregs sharing both phenotypic and functional characteristics with nature Tregs.^27^ However, in the present study, to deplete Tregs, mice were injected intraperitoneally with anti‐CD25 antibody (0.5 mg/d) on days −5, −3 and −1 before testosterone propionate administration and days on 7 and 14 after testosterone propionate administration. After anti‐CD25 antibody administration, we found hyperplasia in the prostate with infiltration of inflammatory cells. The prostatic index of mice was increased after anti‐CD25 antibody administration with significantly increased inflammatory cytokines and PSA secretion and decreased anti‐inflammatory cytokines secretion. These results suggested anti‐CD25 antibody treatment reduce CD4^+^CD25^+^ cells and had few effect on CD4^+^ effector T cells.

The first human clinical trial of adoptively transferred Tregs in Crohn's Disease was reported by Desreumaux et al in 2012.^28^ Marek‐Trzonkowska et al then showed that repetitive administration of polyclonal Tregs was safe and could prolong the survival of β‐cells in patients with type 1 diabetes.^29^ However, a role for Tregs in BPH has been rarely reported. Davidsson et al recently found that high numbers of stromal CD4^+^ Tregs were identified in patients with post‐atrophic hyperplasia and prostate cancer.^10^ In contrast, we found a lower Treg frequency in peripheral blood from BPH patients, but Treg infiltration increased into prostatic tissue with mild or moderate inflammation, and in severely inflamed prostates, Tregs almost disappeared. We hypothesize that different types of patient specimens lead to these inconsistent results. In the study by Davidsson et al,^10^ the prostatic tissues that included prostate cancer, normal tissue, PIN and PAH were all on the same slide, and cancer may have resulted in a higher number of infiltrating CD4^+^ Tregs. In contradistinction, in our study we only recruited patients diagnosed with BPH. In addition, we found that Treg frequency increased in patients with prostate cancer (data not shown).

Emerging studies have shown that Tregs also contribute to repair processes at multiple tissue sites,^30^ including pulmonary repair, neural repair and wound healing.^31^, ^32^, ^33^ Our results are thus consistent with those of previous researchers who indicated that transferred Tregs in BPH mice inhibited prostate inflammation and ameliorated prostate hyperplasia, but also controlled thickening of the bladder's muscular layer. These results may indicate a repair function for Tregs in the prostate and bladder.

The degree of generalizability of our results to BHP patients is unclear owing to the in vivo stability of adoptively transferred Tregs, including their phenotype and function throughout their lifespan. However, excess administration of polyclonal Tregs increases the susceptibility to infection and malignancies.^34^ Nonetheless, adoptive transfer with antigen‐specific Tregs potentially indicates a novel method to prevent and treat BPH.

## CONFLICT OF INTEREST

The authors confirm that there are no conflicts of interest.

## AUTHOR CONTRIBUTION

All authors reviewed the manuscript and approved its content. XJ participated in performing the research, analysing the data and drafting the article. THL conducted the sample collection and immunohistochemistry. GY and HWC performed the histological examination. BT and XYL participated in the ELISA assays. HFL performed flow cytometry. XTC, LNG, HX, YS and PT conducted animal experiments. JQY and HWM performed immunofluorescence staining. JZA, KJW and QW revised the manuscript. LY and HL designed the study and revised the manuscript.

## Supporting information

Table S1‐S2

## Data Availability

This manuscript does not contain sharable data.
