# Supplementary material for: Use of Tregs as a cell‐based therapy via CD39 for benign prostate hyperplasia with inflammation
Source: J Cell Mol Med. 2020 Mar 19;24(9):5082–96. doi: 10.1111/jcmm.15137 (PMC7205803; doi:10.1111/jcmm.15137)
Supplement: Supplementary file 1 — Table S1‐S2 [file JCMM-24-5082-s001.docx]

Supplementary Table 1. Clinical characteristics of patients.

| Patients No. | Age | PSA (ng/ml) | fPSA (ng/ml) | Pathologic diagnosis |
| --- | --- | --- | --- | --- |
| 1 | 70 | 59.03 | 7.8 | Hyperplastic prostate tissue with acute and chronic inflammatory cell infiltration |
| 2 | 81 | 32.51 | 8.24 | Hyperplastic prostate tissue with chronic inflammatory cell infiltration, partially atrophic glands |
| 3 | 66 | 25.42 | 1.73 | Hyperplastic prostate tissue with inflammatory cell infiltration |
| 4 | 76 | 11.27 | 0.75 | Hyperplastic prostate tissue with inflammatory cell infiltration |
| 5 | 54 | 9.46 | 1.51 | Hyperplastic prostate tissue with inflammatory cell infiltration, partially atrophic glands |
| 6 | 61 | 106.76 | 4.40 | Hyperplastic prostate tissue with acute and chronic inflammatory cell infiltration, damaged gland |
| 7 | 65 | 27.21 | 3.48 | Hyperplastic prostate tissue with acute and chronic inflammatory cell infiltration, focal damaged gland |
| 8 | 65 | 10.02 | 1.53 | Hyperplastic prostate tissue with inflammatory cell infiltration |
| 9 | 69 | 13.30 | 2.36 | Hyperplastic prostate tissue with inflammatory cell infiltration, partially atrophic glands |
| 10 | 63 | 1.09 | 0.19 | Hyperplastic prostate tissue with chronic inflammatory cell infiltration |
| 11 | 51 | 6.70 | 1.33 | Hyperplastic prostate tissue with chronic inflammatory cell infiltration |
| 12 | 68 | 9.50 | 0.91 | Hyperplastic prostate tissue with acute and chronic inflammatory cell infiltration, damaged gland |
| 13 | 68 | 35.29 | 5.69 | Hyperplastic prostate tissue with inflammatory cell infiltration, partially atrophic glands |
| 14 | 75 | 7.87 | 2.00 | Hyperplastic prostate tissue with inflammatory cell infiltration |
| 15 | 50 | 22.28 | 2.89 | Hyperplastic prostate tissue with inflammatory cell infiltration, partially atrophic glands |
| 16 | 68 | 11.55 | 2.73 | Hyperplastic prostate tissue with chronic inflammatory cell infiltration, partially atrophic glands |
| 17 | 71 | 79.04 | 30.00 | Hyperplastic prostate tissue with acute and chronic inflammatory cell infiltration, damaged gland |
| 18 | 81 | 49.50 | 2.85 | Hyperplastic prostate tissue with acute and chronic inflammatory cell infiltration, damaged gland |
| 19 | 57 | 5.11 | 0.61 | Hyperplastic prostate tissue with inflammatory cell infiltration |
| 20 | 60 | 5.82 | 0.85 | Hyperplastic prostate tissue with inflammatory cell infiltration |
| 21 | 78 | 17.65 | 4.26 | Hyperplastic prostate tissue |
| 22 | 62 | 6.82 | 1.50 | Hyperplastic prostate tissue |
| 23 | 64 | 11.96 | 0.48 | Hyperplastic prostate tissue with partially atrophic glands |
| 24 | 52 | 9.33 | 0.53 | Hyperplastic prostate tissue |
| 25 | 76 | 2.47 | 0.63 | Hyperplastic prostate tissue |
| 26 | 79 | 3.18 | 0.82 | Hyperplastic prostate tissue |
| 27 | 66 | 18.67 | 2.66 | Hyperplastic prostate tissue |
| 28 | 69 | 3.85 | 1.52 | Hyperplastic prostate tissue |
| 29 | 64 | 22.87 | 2.49 | Hyperplastic prostate tissue |
| 30 | 69 | 8.29 | 1.74 | Hyperplastic prostate tissue |
| 31 | 70 | 15.39 | 3.03 | Hyperplastic prostate tissue |
| 32 | 68 | 16.33 | 0.85 | Hyperplastic prostate tissue |
| 33 | 72 | 9.67 | 0.738 | Hyperplastic prostate tissue |
| 34 | 65 | 11.94 | 2.9 | Hyperplastic prostate tissue |
| 35 | 77 | 10.83 | 11.73 | Hyperplastic prostate tissue |
| 36 | 59 | 8.12 | 1.1 | Hyperplastic prostate tissue |
| 37 | 64 | 14.81 | 2.2 | Hyperplastic prostate tissue |
| 38 | 75 | 5.15 | 1.95 | Hyperplastic prostate tissue |

Supplementary Table 2. Phenotype characteristics of CD39^+/-^ Tregs in prostate and peripheral blood after Tregs infusion.

| Prostate | (%) | CTLA-4 | Foxp3 | CD62L | LAG-3 | CD44^+^CCR7^+^ | CD44^+^CCR7^-^ | CD44^-^CCR7^+^ |
| --- | --- | --- | --- | --- | --- | --- | --- | --- |
| Transfer of CD39^+^Treg | CD39^+^ (72.25±6.15) | 91.80±1.70 | 60.20±5.10 | 67.65±4.35 | 36.30±4.70 | 32.00±2.80 | 40.35±10.35 | 10.12±3.18 |
|  | CD39^-^ (24.35±6.15) | 25.80±0.80 | 33.45±6.05 | 4.66±0.55 | 6.54±0.66 | 5.37±0.11 | 11.65±0.65 | 34.45±2.55 |
| Transfer of CD39^-^Treg | CD39^+^( 45.15±1.95) | 84.45±7.05 | 27.45±0.35 | 61.90±6.90 | 38.00±1.90 | 20.50±5.20 | 46.53±11.30 | 9.92±2.98 |
|  | CD39^-^ (50.45±2.05) | 28.15±2.50 | 25.80±1.10 | 2.53±0.18 | 1.27±0.38 | 0.00±0.00 | 6.91±0.15 | 10.63±1.17 |
| Transfer of CD25^+^Treg | CD39^+^ (64.50±3.40） | 93.35±1.65 | 33.70±8.80 | 49.00±3.50 | 21.56±14.63 | 29.55±3.35 | 50.00±2.50 | 6.85±0.66 |
|  | CD39^-^ (32.91±5.30) | 53.10±1.80 | 30.45±2.45 | 1.61±0.39 | 5.92±4.09 | 3.66±3.66 | 16.00±6.00 | 16.70±0.70 |
| Peripheral blood | (%) | CTLA-4 | Foxp3 | CD62L | LAG-3 | CD44^+^CCR7^+^ | CD44^+^CCR7^-^ | CD44^-^CCR7^+^ |
| Transfer of CD39^+^Treg | CD39^+^ (59.37±3.14) | 43.65±0.65 | 50.10±1.20 | 85.80±1.30 | 77.25±0.50 | 9.99±0.51 | 6.21±0.10 | 52.95±1.50 |
|  | CD39^-^ (36.20±4.18) | 39.55±1.25 | 59.15±0.75 | 59.00±0.25 | 63.45±1.55 | 1.38±0.35 | 1.46±0.05 | 53.60±1.14 |
| Transfer of CD39^-^Treg | CD39^+^ (45.66±7.62) | 46.15±3.35 | 49.75±1.75 | 85.65±1.15 | 72.60±1.90 | 8.56±1.65 | 8.06±0.33 | 46.55±1.55 |
|  | CD39^-^ (49.53±9.48) | 30.03±1.00 | 68.40±1.50 | 52.70±1.90 | 58.65±1.95 | 1.94±0.12 | 1.15±0.27 | 49.55±0.45 |
| Transfer of CD25^+^Treg | CD39^+^ (51.10±1.96) | 44.20±5.10 | 41.10±2.70 | 79.45±2.50 | 77.10±2.60 | 9.21±0.16 | 5.99±0.20 | 52.85±3.20 |
|  | CD39^-^ (44.9±1.76) | 32.10±10.01 | 60.00±1.00 | 45.80±2.80 | 67.90±3.70 | 0.88±0.18 | 0.80±0.27 | 59.60±7.00 |

Tregs were gated on CD4^+^CD25^+^ cells, and then phenotype characteristics were gated on CD39^+/-^Tregs. In the CD39^+/-^Treg cell subsets, resting Tregs were gated as CD44^−^CCR7^+^, effector Tregs were gated as CD44^+^CCR7^−^, and memory Tregs were gated as CD44^+^CCR7^+^ cells.
